# A New Reassigned Spectrogram Method in Interference Detection for GNSS Receivers

**DOI:** 10.3390/s150922167

**Published:** 2015-09-02

**Authors:** Kewen Sun, Tian Jin, Dongkai Yang

**Affiliations:** 1 School of Computer and Information, Hefei University of Technology, Tunxi Road 193, Hefei 230009, China; 2 School of Electronic and Information Engineering, Beihang University, XueYuan Road 37, Beijing 100191, China; E-Mails: jintian@buaa.edu.cn (T.J.); edkyang@buaa.edu.cn (D.Y.)

**Keywords:** Global Navigation Satellite System (GNSS), interference detection and mitigation, spectrogram, Wigner–Ville distribution (WVD), reassigned method, notch filter

## Abstract

Interference detection is very important for Global Navigation Satellite System (GNSS) receivers. Current work on interference detection in GNSS receivers has mainly focused on time-frequency (TF) analysis techniques, such as spectrogram and Wigner–Ville distribution (WVD), where the spectrogram approach presents the TF resolution trade-off problem, since the analysis window is used, and the WVD method suffers from the very serious cross-term problem, due to its quadratic TF distribution nature. In order to solve the cross-term problem and to preserve good TF resolution in the TF plane at the same time, in this paper, a new TF distribution by using a reassigned spectrogram has been proposed in interference detection for GNSS receivers. This proposed reassigned spectrogram method efficiently combines the elimination of the cross-term provided by the spectrogram itself according to its inherent nature and the improvement of the TF aggregation property achieved by the reassignment method. Moreover, a notch filter has been adopted in interference mitigation for GNSS receivers, where receiver operating characteristics (ROCs) are used as metrics for the characterization of interference mitigation performance. The proposed interference detection method by using a reassigned spectrogram is evaluated by experiments on GPS L1 signals in the disturbing scenarios in comparison to the state-of-the-art TF analysis approaches. The analysis results show that the proposed interference detection technique effectively overcomes the cross-term problem and also keeps good TF localization properties, which has been proven to be valid and effective to enhance the interference detection performance; in addition, the adoption of the notch filter in interference mitigation has shown a significant acquisition performance improvement in terms of ROC curves for GNSS receivers in jamming environments.

## 1. Introduction

Recently, the demand for safety-critical applications (e.g., civil aviation, aircraft landing) using Global Navigation Satellite Systems (GNSSs) has gained extensive and increased interest. With the development of the new GNSSs, such as the European Union’s Galileo system [1], the U.S.’s modernized GPS [2] and China’s Beidou/Compass [3], more efforts have been devoted to the design of high performance GNSS receivers, which are expected to increase the accuracy, availability, integrity and continuity of service, especially in the field of safety of life (SOL) applications (e.g., the accuracy needed during the landing of an aircraft). High precision positioning and reliable SOL services represent the main challenges for the upcoming satellite navigation systems. The vulnerability of GNSS receivers to interference has become an increasingly important issue. The direct sequence spread spectrum (DSSS), used in most of the satellite navigation systems, spreads the received GNSS signal power over a wider bandwidth; this ensures a dispreading gain in the GNSS receiver, which can reduce impairments caused by the undesired disturbing signals. Although GNSS has a certain capability to be immune from interference, since DSSS is utilized, due to the reception of the very low GNSS signal power, the presence of intentional or unintentional disturbing signals, such as spurious, harmonics and electromagnetic interferences, will result in serious performance degradation of GNSS receivers, including the inability to determine the correct position.

Among all of the different error sources that can potentially corrupt GNSS signals, radio frequency interference (RFI) is particularly harmful, since, in some cases, it cannot be mitigated by a simple correlation process [4]. The jamming environment is threatening for satellite navigation systems. Many systems rely on the transmission of radio frequency (RF) energy in the L-band. For example, the European Galileo E5a and E5b radio bands, located within 1164–1214 MHz, occupy frequencies already allocated for aeronautical radio navigation services (ARNS), such as tactical air navigation (TACAN), distance measuring equipment (DME) and secondary surveillance radar (SSR) [5]. In addition, illegal portable jamming devices are becoming popular to protect traffic drivers from being tracked by GNSS receivers in their vehicles. These so-called personal privacy devices radiate disruptive interfering signals in the GNSS frequency bands, which can make a conventional GNSS receiver inoperable. The interference impairments can heavily degrade the reception of useful GNSS signals and deny the GNSS-based services in a geographical area with a radius of several kilometers [6]. It emerges that interfering signals are expected to become a very serious threat for satellite navigation operations, and countermeasures have to be taken to prevent interference from blocking the GNSS receivers, especially for safety-critical applications.

In the literature, several interference detection and mitigation techniques have been proposed and investigated, and each of them differs according to the operation domain (time, frequency or space). These techniques can be classified according to the specific processing domain. In the time domain techniques, temporal filtering can be usually adopted to suppress temporally-correlated (narrowband) interference, since wideband interference cannot be easily discriminated from thermal noise. Frequency domain techniques are generally based on spectral estimation of the incoming signal, which is obtained by applying signal processing techniques, such as discrete Fourier transform (DFT). These frequency domain techniques are typically performed by comparing the spectrum of the received signal with a theoretical threshold, which is usually determined according to a statistical model representing the received signal [7]. In addition, spatial filtering is often used in the antenna array receivers for interference suppression [8]. The disadvantage of spatial filtering is that the degrees of freedom are limited by the number of antenna elements; a spatial filter with a limited number of degrees of freedom is not able to sharply separate the received signal directions. In this way, the mitigation of the interfering signal in the spatial domain also results in the suppression of the useful GNSS signals, particularly when they have a direction close to that of the interfering signals.

Nowadays, the research topic on time-frequency (TF) transforms adopted in interference detection for GNSS receivers has obtained increasing attention [9,10]. In many cases, interferences may appear for a limited time and present a very variable behavior in frequency. In comparison to the GNSS signal, interfering signals are extremely different in terms of time and frequency characteristics. Interfering signals usually present a sharp TF energy concentration in a limited region of the two-dimensional TF plane, while the GNSS signal and noise term spread over the entire TF plane. Since the TF characteristics of the interference can be easily distinguished from those of the useful GNSS signal and the noise term in the TF plane, the TF analysis techniques can be used as very effective countermeasures for detecting a large variety of interfering signals in GNSS receivers. Different TF representations (TFRs), such as the spectrogram and the Wigner–Ville distribution (WVD), have been considered in interference detection for GNSS applications [9,10]. The spectrogram can be generated via a moving window in time or a moving window in frequency, and its characteristics strictly depend on the used analysis window function. The spectrogram approach presents a trade-off between the time and frequency resolutions as a consequence of Heisenberg’s uncertainty principle, providing poor TF localization properties. In order to deal with the TF resolution limitation present in the spectrogram, WVD has been used in interference detection for GNSS receivers [9,10]. WVD is well known, since it provides nearly the best TF resolution among all of the TF distributions and also satisfies a large number of desirable theoretical properties, but it presents very severe cross-interfering terms without any physical meaning between true signal components (auto-terms), due to the interaction of different frequency components [11,12]. The presence of cross-terms leads to a distorted interpretation in the TF plane, which makes the TF transform difficult to visually interpret and results in serious instantaneous frequency estimation error for the interfering signals. The WVD approach is seriously impaired by the cross-term problem, which limits the effectiveness of its sharp localization in multi-component signal situations. Therefore, a total or partial removal of such parasite terms of the bilinear TF distributions is required.

In order to overcome or attenuate the cross-interfering terms present in the the quadratic TF distributions, the Choi–Williams transform (CWT) has been proposed to detect the sweep interference for GNSS receivers, but the cross-term attenuation with the CWT approach is paid at the price of TF resolution degradation [10]. Additionally, several kernel design methods have been proposed to mitigate the cross-term effect [13,14,15]; unfortunately, these techniques need heavy computational complexity when they are applied in a real-time context. The unsatisfactory results obtained with the existing TF distributions justify the search for better tools; one way of achieving this is to start from the general form of quadratic representations. All of the TF distributions of current interest could be subsumed in the general framework of Cohen’s class, whose element can be written as the generalized filtered version of the WVD with a two-dimensional smoothing kernel [11]. This smoothing operation is able to mitigate the cross-terms present in the TF distributions; however, it also produces a less accurate TF localization of the signal components. Therefore, a suitable trade-off should be found between the attenuation of the misleading cross-terms and the TF energy concentration of the signal components.

A meaningful solution to deal with this trade-off for the TF distributions is the reassignment method [16,17,18], which is a point-wise transformation of regular TFRs; it works by relocating the values of a TFR from the geometrical center of the analysis window to the center of gravity, which is more representative of the localization of the TF energy distribution. In this way, the reassignment method smooths out oscillating cross-terms while squeezing localized terms, which spread over the TF plane; and the TF energy density components are relocated to the position causing the signal features. The main feature of the reassigned TF distribution is that it provides good TF localization properties. Since the spectrogram is a typical type of cross-term-free representation of Cohen’s class, in this paper, a modified version of the spectrogram based on the reassignment in the TF plane has been applied in interference detection for GNSS receivers in the jamming scenarios. The reassigned spectrogram allows improving the TF resolution to provide a readable and localized distribution in the TF domain. This results in a squeezed representation with sharp TF energy concentration, which presents no cross-terms in the TF plane.

The motivation of this paper is to design a novel TF distribution for the removal of the serious cross-terms and the enhancement of TF resolution, which is suitable to be used in interference detection for GNSS receivers. Therefore, in this paper, a novel TF analysis method by adopting a reassigned spectrogram has been proposed in interference detection for GNSS receivers. To the best of our knowledge, this interference detection technique based on the reassigned spectrogram in the interference detection units for GNSS receivers is new. The performance of the proposed method has been deeply evaluated in comparison to the existing TF analysis approaches. Different localization properties and cross-term effects in the TF plane have been well investigated and compared among the aforementioned TF distributions adopted in interference detection for GNSS receivers.

In addition, the acquisition performances in the presence and absence of interference mitigation units in GNSS receivers have been studied. In this paper, the interference is mitigated by using a notch filter, which is implemented by an impulse response filter (IIR) based on the interference instantaneous frequency estimated by the proposed reassigned spectrogram method. The interference mitigation performance has been evaluated in terms of the receiver operating characteristic (ROC) or the ROC curve by means of Monte Carlo simulations [19,20,21]. The ROC curve is a graphical plot of the sensitivity, plotting the behaviors of the detection probability $P_{d}$ versus the false alarm probability $P_{fa}$ or, equivalently, of the missed detection probability $P_{m}$ versus the false alarm probability $P_{fa}$ of a binary classifier system as its discrimination threshold is varied. Therefore, the ROC curve can be used to completely characterize the acquisition performance, which provides a statistical characterization of the acquisition performance allowing comparative analysis for different algorithms.

Among all of the different classes of interfering signals, a constant amplitude linearly-modulated sweep interference (chirp disturbance) has been shown to have severe impacts on the quality of the received GNSS signal [6,10,22]. Therefore, in this paper, such an interference has been employed as a test bench for the the proposed TF analysis method. In order to prove the effectiveness of the proposed reassigned spectrogram in interference detection for GNSS receivers, an experiment has been accomplished in the GPS L1 signal, which is characterized in additive white Gaussian noise (AWGN) corrupted by chirp interference. The analysis results show that the proposed TF analysis by using reassigned spectrogram eliminates the cross-term artifacts present in the quadratic TF distribution and presents good resolution of time and frequency in the TF plane during the identification and detection of interfering signals for GNSS receivers. This developed TF analysis technique by using a reassigned spectrogram in interference detection makes the TF characteristics of the interfering term sharply distinguishable among the received GNSS signal, which provides improved readability and localization properties in the TF plane and presents enhanced interference detection performance for GNSS receivers with respect to the state-of-the-art TF analysis approaches. In particular, the ROC curves have been adopted as metrics for verifying the effectiveness of the interference mitigation technique for GNSS receivers. The adoption of the notch IIR filter in interference mitigation for GNSS receivers has led to a significant acquisition performance improvement in the disturbing scenarios.

## 2. Signal and System Model

The signal at the input of a GNSS receiver, in a noisy environment with RFI, can be written as:

(1) $$\begin{array}{c} y_{RF}\left(t\right)=\sum_{i=1}^{N_{s}}r_{RF,i}\left(t\right)+\eta_{RF}\left(t\right) \end{array}$$

*i.e.*, the sum of $N_{s}$ useful signals emitted by $N_{s}$ different satellites and of a disturbing term $\eta_{RF}\left(t\right)$, and $N_{s}$ is the number of satellites in view. The expression of the signal in space (SIS) transmitted by the *i*-th satellite and received at the GNSS receiver antenna with a propagation delay $\tau_{i}$ is usually assumed as the following structure:

(2) $$\begin{array}{c} r_{RF,i}\left(t\right)=A_{i}c_{i}\left(t-\tau_{i}\right)d_{i}\left(t-\tau_{i}\right)\cos\left[2\pi \left(f_{RF}+f_{d,i}\right)t+\varphi_{RF,i}\right] \end{array}$$

where:

- $A_{i}$ is the amplitude of the *i*-th useful signal;
- $\tau_{i}$ is the code phase delay introduced by the transmission channel;
- $c_{i}\left(t-\tau_{i}\right)$ is the pseudo random noise (PRN) code sequence, which is assumed to take a value in the set {−1,1};
- $d_{i}\left(t-\tau_{i}\right)$ is the bit stream of the navigation message, binary phase-shift keying (BPSK) modulated, including satellite data; and each binary unit is called a bit;
- $f_{d,i}$ is the Doppler frequency shift affecting the *i*-th useful signal, and $\varphi_{RF,i}$ is the initial carrier phase offset;
- $f_{RF}$ is the carrier frequency, and it depends on the GNSS signal band under analysis; in the case of the GPS L1 signal, $f_{RF}=f_{L}1=1,575.42$ MHz.

In general, the disturbing term $\eta_{RF}\left(t\right)$ can be expressed as:

(3) $$\begin{array}{c} \eta_{RF}\left(t\right)=j_{RF}\left(t\right)+w_{RF}\left(t\right) \end{array}$$

where $j_{RF}\left(t\right)$ is a non-stationary RFI and $w_{RF}\left(t\right)$ is a zero-mean stationary AWGN process.

The interfering signal $j_{RF}\left(t\right)$ can assume different forms depending on the jammer that generates it. Several efforts have been devoted to the analysis and characterization of civilian GNSS jammers; despite significant differences, the transmitted jamming signal is usually frequency modulated with an almost constant amplitude. In this paper, the interference term $j_{RF}\left(t\right)$ is assumed to be in the class of sweep interference (linear chirp). Sweep interference is one of the main classes of the interfering signals, and its corresponding time domain function represented by sinusoids can be written as follows:

(4) $$\begin{array}{c} j_{RF}\left(t\right)=A_{inst}\left(t\right)\cos\left[2\pi f_{inst}\left(t\right)t+\varphi_{0}\right] \end{array}$$

where $A_{inst}\left(t\right)$ is the interfering signal amplitude, $f_{inst}\left(t\right)$ denotes its instantaneous frequency and $\varphi_{0}$ is the initial phase of the interference (at time t=0), which can be assumed to be a random variable with a uniform distribution in the range [−π,+π).

In a linear chirp, the instantaneous frequency $f_{inst}\left(t\right)$ of the interfering signal evolves linearly with time over the interval $\left[f_{RF}+\Delta f_{0},f_{RF}+\Delta f_{1}\right]$, where $f_{RF}$ is the GNSS signal center frequency. Therefore, the instantaneous frequency $f_{inst}\left(t\right)$ can be expressed as:

(5) $$\begin{array}{c} f_{inst}\left(t\right)=f_{0}+kt\;\;\;\;\;0\leq t\leq t_{j} \end{array}$$

where $f_{0}$ is the starting frequency (at time t=0), $f_{0}=f_{RF}+\Delta f_{0}$, $t_{j}$ is the frequency sweep period of the jamming signal and *k* is the rate of frequency increase or chirp rate, written as:

(6) $$\begin{array}{c} k=\frac{f_{1}-f_{0}}{t_{j}}=\frac{\Delta f_{1}-\Delta f_{0}}{t_{j}} \end{array}$$

where $f_{1}$ is the final frequency during a specific frequency sweep period, $f_{1}=f_{RF}+\Delta f_{1}$ and $\Delta f_{1}-\Delta f_{0}$ stands for the frequency sweep.

The input signal $y_{RF}\left(t\right)$ defined in Equation (1) is received by the receiver antenna, down-converted and filtered by the receiver front-end. Then, the received signal before the analog-to-digital (A/D) conversion can be written as:

(7) $$\begin{array}{c} \begin{array}{cc} y(t)= & \sum_{i=1}^{N_{s}}r_{i}\left(t\right)+\eta \left(t\right)\\ = & \sum_{i=1}^{N_{s}}A_{i}{\tilde{c}}_{i}\left(t-\tau_{i}\right)d_{i}\left(t-\tau_{i}\right)\cos\left[2\pi \left(f_{IF}+f_{d,i}\right)t+\varphi_{i}\right]+\eta \left(t\right) \end{array} \end{array}$$

where $f_{IF}$ is the receiver intermediate frequency (IF). The term ${\tilde{c}}_{i}\left(t-\tau_{i}\right)$ represents the spreading sequence after filtering of the front-end, and here, the simplifying condition:

(8) $$\begin{array}{c} {\tilde{c}}_{i}\left(t\right)\approx c_{i}\left(t\right) \end{array}$$

is assumed and the impact of the front-end filter is neglected. η(t) is the down-converted and filtered disturbing component, η[t]=j[t]+w[t].

Considering the interference term j[t], the mean power of the sweep interference can be defined as:

(9) $$\begin{array}{c} J=\mathrm{Var}\{j[t]\} \end{array}$$

The jammer-to-noise ratio (JNR) is defined as follows:

(10) $$\begin{array}{c} \frac{J}{N}=\frac{J}{\sigma_{IF}^{2}}=\frac{J}{N_{0}B_{IF}} \end{array}$$

where $\sigma_{IF}^{2}$ is the variance of the IF noise, $N_{0}/2$ is the power spectral density of the IF noise and $B_{IF}$ is the front-end bandwidth.

In order to avoid the cross-terms resulting from the interaction between the positive and negative frequency parts of the spectrum, the analytic representation of the received signal is adopted, provided as follows:

(11) $$\begin{array}{c} y_{a}\left[t\right]=y\left[t\right]+j\overset{˰}{y}\left[t\right] \end{array}$$

where the analytic signal $y_{a}\left[t\right]$ has a real part y[t] and an imaginary part $\overset{˰}{y}\left[t\right]$, which contains the Hilbert transform of y[t]. The imaginary part is a version of the original real part with a $90^{\circ }$ phase shift. The use of the analytic signal has two advantages: first, interference between positive and negative frequencies can be eliminated, as the analytic signal $y_{a}\left[t\right]$ has components belonging only to the half plane of positive frequencies; second, even though this filtering suppresses negative frequencies and the sampling rate is reduced, it does not introduce any loss of information.

## 3. Time Frequency Transforms

The classical method for analyzing a signal with time-varying frequency content is to split the time domain signal into many segments. The signal to be transformed is multiplied by a window function, which is nonzero for only a short period of time, and, then, taking the Fourier transform of each segment as the window slid along the time axis, resulting in a two-dimensional representation of the signal. This is known as the short-time Fourier transform (STFT) operation, which is the most widely-used method for analyzing non-stationary signals. Additionally, simply, in the continuous time case, it is defined as:

(12) $$\begin{array}{c} STFT\left(t,\omega \right)=\int_{-\infty }^{+\infty }y_{a}\left(\tau \right)h\left(\tau -t\right)e^{-j\omega \tau }\text{d}\tau \end{array}$$

where h(t) is the analysis window function, which is a real and even window function centered on zero, and the commonly-used window functions include Hamming, Hanning, Chebyshev, Kaiser and Gaussian windows; $y_{a}\left(t\right)$ is the defined analytical signal to be transformed, and it is fed through the analysis bandpass filter with center frequency ω whose bandwidth is equal to that of the analysis window *h*; and STFT(t,ω) is a function of *t* and ω, which is linear and depends on the chosen window *h*.

This operation in Equation (12) differs from the classical Fourier transform only by the presence of a window function h(t). The STFT is generated by taking the Fourier transform of smaller durations of the original signal. Alternatively, the STFT can be interpreted as the projection of the analytic signal $y_{a}\left(\tau \right)$ onto a set of bases $h^{*}\left(\tau -t\right)exp\left\{j\omega \tau \right\}$ with parameters *t* and ω. Since the bases are no longer of infinite extent in time, it is possible to monitor how the signal frequency spectrum varies as a function of time. This is accomplished by the translation of the window as a function of time instant *t*, resulting in a two-dimensional joint TFR STFT(t,ω) of the original time signal.

In order to understand the time properties at a particular frequency, the definition of the STFT can also be expressed in the frequency domain by manipulating Equation (12), obtaining the following result:

(13) $$\begin{array}{c} STFT\left(t,\omega \right)=\frac{1}{2\pi }e^{-j\omega t}\int_{-\infty }^{+\infty }Y_{a}\left(\omega^{\prime }\right)H\left(\omega^{\prime }-\omega \right)e^{j\omega^{\prime }t}\text{d}\omega^{\prime } \end{array}$$

where H(ω) is the frequency window function, which is the Fourier transform of h(t). The dual relationship between Equations (12) and (13) is apparent; the TFR can be generated via a moving window in time or a moving window in frequency.

In the STFT analysis, one intends to achieve both high time and frequency resolutions if possible. However, the resolution in the time domain is limited by the width of the window function h(t); similarly, the resolution in the frequency domain is limited by the width of the frequency window H(ω). As a result, the choice of a window to represent the signal by the STFT imposes a compromise between the conservation of temporal localization and that of frequency localization. This compromise is due to Heisenberg’s uncertainty principle, which states that the time and frequency standard deviations of the corresponding window functions are related by the following inequality:

(14) $$\begin{array}{c} \sigma_{t}\sigma_{\omega }\geq \frac{1}{2} \end{array}$$

where:

(15) $$\begin{array}{c} \sigma_{t}={\left[\frac{\int_{-\infty }^{+\infty }{\left(t-\mu_{t}\right)}^{2}{\left|h(t)\right|}^{2}\text{d}t}{\int_{-\infty }^{+\infty }{\left|h(t)\right|}^{2}\text{d}t}\right]}^{1/2} \end{array}$$

(16) $$\begin{array}{c} \sigma_{\omega }={\left[\frac{\int_{-\infty }^{+\infty }{\left(\omega -\mu_{\omega }\right)}^{2}{\left|H(\omega )\right|}^{2}\text{d}\omega }{\int_{-\infty }^{+\infty }{\left|H(\omega )\right|}^{2}\text{d}\omega }\right]}^{1/2} \end{array}$$

and the mean time $\mu_{t}$ and mean frequency $\mu_{\omega }$ are defined as follows:

(17) $$\begin{array}{c} \mu_{t}=\frac{\int_{-\infty }^{+\infty }t{\left|h(t)\right|}^{2}\text{d}t}{\int_{-\infty }^{+\infty }{\left|h(t)\right|}^{2}\text{d}t} \end{array}$$

(18) $$\begin{array}{c} \mu_{\omega }=\frac{\int_{-\infty }^{+\infty }\omega {\left|H(\omega )\right|}^{2}\text{d}\omega }{\int_{-\infty }^{+\infty }{\left|H(\omega )\right|}^{2}\text{d}\omega } \end{array}$$

From Equation (14), it is easy to know that the window width in time and the window width in frequency are inversely proportional to each other. Therefore, choosing a small time window leads to good resolution in time and necessarily implies poor resolution in frequency; conversely, a long time window yields poor time resolution, but good frequency resolution can be obtained. The length of the window function plays a fundamental role in this TF compromise.

The squared magnitude of the STFT, denoted by S(t,ω), is called the spectrogram, which can be written as follows:

(19) $$\begin{array}{c} S\left(t,\omega \right)={\left|STFT\left(t,\omega \right)\right|}^{2} \end{array}$$

where STFT(t,ω) is the STFT defined in Equation (12). The spectrogram of the signal has poor TF localization properties due to the presence of the analysis window function.

**Figure 1:**
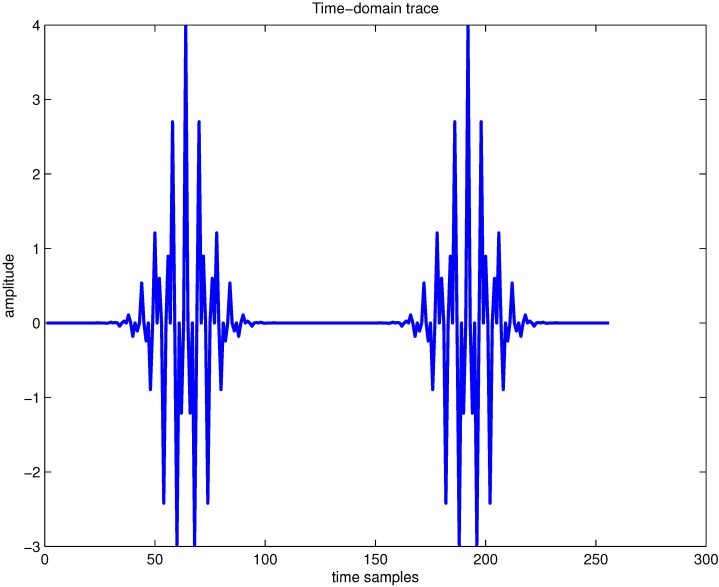
Time domain trace of four Gaussian components’ signal.

**Figure 2:**
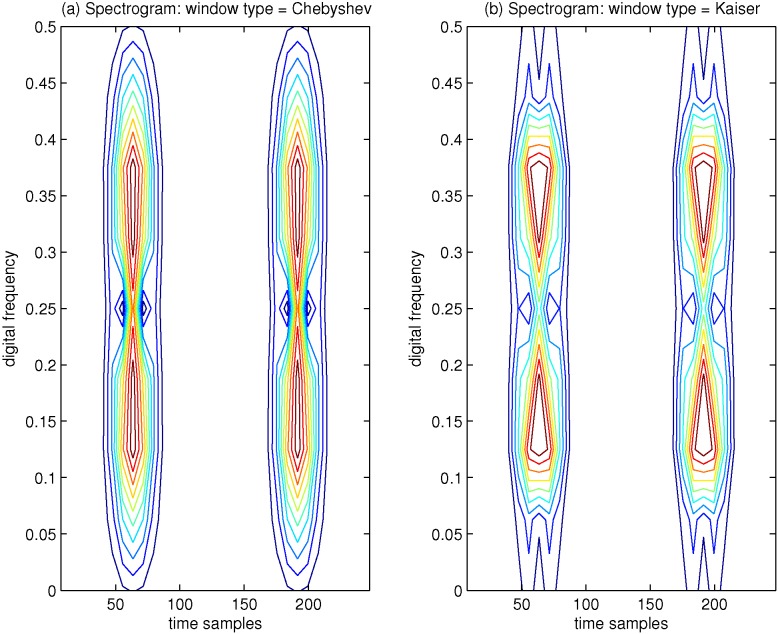
Spectrogram of four Gaussian components’ signals, which has been calculated by using a window length of 15 samples. (**a**) Window type = Chebyshev; (**b**) Window type = Kaiser.

Here, we illustrate the influence effect on the spectrogram by using different types and lengths of the analysis window functions. As an example, the time domain signal, which contains four Gaussian components, is shown in Figure 1. By using different types of analysis window functions, the spectrogram of this signal is provided in Figure 2. In Figure 2a, the Chebyshev window function is selected, and in Figure 2b, the Kaiser window function is used; in both cases, the window size is 15 samples. It is clear that the spectrogram shows poor TF localization properties, which presents different TF characteristics depending on the selected window type.

**Figure 3:**
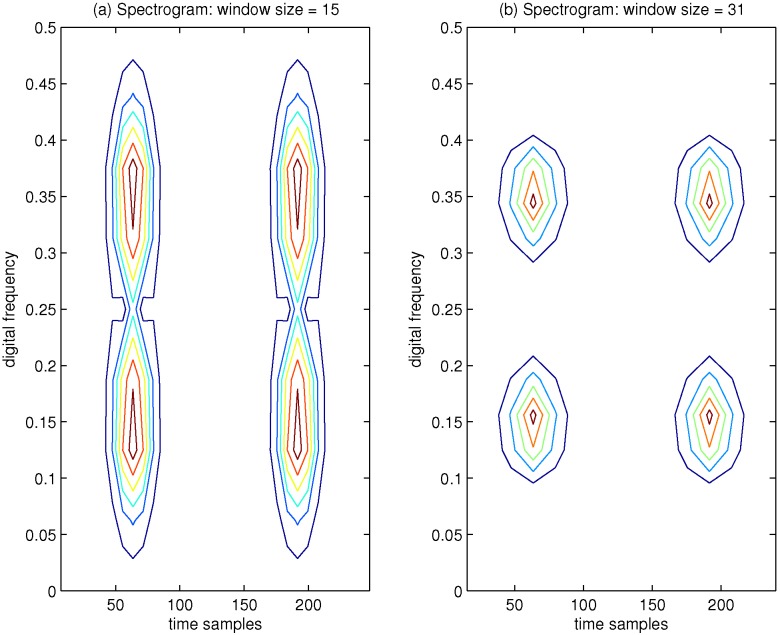
Spectrogram of four Gaussian components’ signals, which has been calculated by using Hamming window function. (**a**) Window size = 15; (**b**) Window size = 31.

Furthermore, in order to evaluate the TF characteristics of the spectrogram by varying the length of the analysis window function, an example of the spectrogram of the aforementioned signal is presented in Figure 3, where the Hamming window is adopted. In Figure 3a, the window size is 15 samples, and it is easy to know that the small time window length implies poor resolution in frequency; in Figure 3b, the window size is increased to 31 samples, and improved resolution in frequency can be achieved when the window length is increased. From Figure 3, we can conclude that if the length of the adopted window function is small, then the localization of the frequency content of the aforementioned signal will be smeared and vice versa. This further verifies the limitation from the uncertainty principle with the spectrogram approach. We can know that the spectrogram of the signal presents the TF resolution trade-off problem due to the presence of the analysis window function.

In order to avoid the TF resolution trade-off problem of the spectrogram, WVD is adopted in interference detection for GNSS receivers. In the WVD, a time-dependent instantaneous auto-correlation function is chosen as:

(20) $$\begin{array}{c} R\left(t,\tau \right)=y_{a}\left(t+\frac{\tau }{2}\right)y_{a}^{*}\left(t-\frac{\tau }{2}\right) \end{array}$$

The WVD of $y_{a}\left(t\right)$ is then defined as the Fourier transform of this time-dependent instantaneous auto-correlation function [23], written as follows:

(21) $$\begin{array}{c} WVD\left(t,\omega \right)=\frac{1}{2\pi }\int_{-\infty }^{+\infty }R\left(t,\tau \right)e^{-j\omega \tau }\text{d}\tau =\frac{1}{2\pi }\int_{-\infty }^{+\infty }y_{a}\left(t+\frac{\tau }{2}\right)y_{a}^{*}\left(t-\frac{\tau }{2}\right)e^{-j\omega \tau }\text{d}\tau \end{array}$$

where $y_{a}\left(\cdot \right)$ denotes the signal to be analyzed, *t* is the time, ω represents the angular frequency, τ is called the lag variable and (*) denotes the complex conjugate.

In addition, this class of bilinear (or quadratic) TF distributions can be most easily understood in terms of the ambiguity function. If the inverse Fourier transform of the instantaneous auto-correlation function R(t,τ) is taken with respect to *t* instead of τ, the ambiguity function can be obtained as follows:

(22) $$\begin{array}{c} AF\left(\tau ,\theta \right)=\int_{-\infty }^{+\infty }R\left(t,\tau \right)e^{j\theta t}\text{d}t=\int_{-\infty }^{+\infty }y_{a}\left(t+\frac{\tau }{2}\right)y_{a}^{*}\left(t-\frac{\tau }{2}\right)e^{j\theta t}\text{d}t \end{array}$$

The ambiguity function can be used to monitor the disturbing effect in the received GNSS signals.

The WVD has a number of desirable properties that make it a good indicator of how the energy of the signal can be viewed as a function of time and frequency. First, the WVD of any signal is always real. Second, it satisfies the time marginal condition:

(23) $$\begin{array}{c} \int_{-\infty }^{+\infty }WVD\left(t,\omega \right)\text{d}\omega ={\left|y_{a}\left(t\right)\right|}^{2} \end{array}$$

That is, by summing the TF distribution over all frequencies, the instantaneous energy of the signal at a particular time instant can be obtained. Similarly, the WVD also satisfies the frequency marginal condition:

(24) $$\begin{array}{c} \int_{-\infty }^{+\infty }WVD\left(t,\omega \right)\text{d}t={\left|Y_{a}\left(\omega \right)\right|}^{2} \end{array}$$

In this case, by summing the TF distribution over all time, the power spectrum of the signal at a particular frequency can be obtained.

Although WVD has many good properties and provides nearly the best resolution among all of the TF techniques, its main drawback comes from undesirable cross-term interference. The WVD is said to be bilinear, because the analyzed signal enters twice in its calculation. Consider the signal $y\left(t\right)=y_{1}\left(t\right)+y_{2}\left(t\right)$, where y(t), $y_{1}\left(t\right)$ and $y_{2}\left(t\right)$ are analytic. Expanding the instantaneous auto-correlation function of y(t), we can obtain:

(25) $$\begin{array}{c} R_{y}\left(t,\tau \right)=R_{y_{1}}\left(t,\tau \right)+R_{y_{2}}\left(t,\tau \right)+R_{y_{1}y_{2}}\left(t,\tau \right)+R_{y_{2}y_{1}}\left(t,\tau \right) \end{array}$$

where $R_{y_{1}y_{2}}\left(t,\tau \right)$ and $R_{y_{2}y_{1}}\left(t,\tau \right)$ are the instantaneous cross-correlation functions (e.g., $R_{y_{1}y_{2}}\left(t,\tau \right)=y_{1}\left(t+\frac{\tau }{2}\right)y_{2}^{*}\left(t-\frac{\tau }{2}\right)$). Taking the Fourier transforms of Equation (25) with respect to τ, it is easy to know that:

(26) $$\begin{array}{cc} WVD_{y}\left(t,\omega \right) & \;\;\;\;=WVD_{y_{1}}\left(t,\omega \right)+WVD_{y_{2}}\left(t,\omega \right)+2Re\left\{WVD_{y_{1}y_{2}}\left(t,\omega \right)\right\} \end{array}$$

where $WVD_{y_{1}}\left(t,\omega \right)$ and $WVD_{y_{2}}\left(t,\omega \right)$ are the WVDs of $y_{1}\left(t\right)$ and $y_{2}\left(t\right)$, respectively, and the last term is the cross-WVD (XWVD) between $y_{1}\left(t\right)$ and $y_{2}\left(t\right)$, provided as:

(27) $$\begin{array}{c} WVD_{y_{1}y_{2}}\left(t,\omega \right)=\frac{1}{2\pi }\int_{-\infty }^{+\infty }y_{1}\left(t+\frac{\tau }{2}\right)y_{2}^{*}\left(t-\frac{\tau }{2}\right)e^{-j\omega \tau }\text{d}\tau \end{array}$$

Thus, the WVD of the sum of two signals is not the sum of their corresponding WVDs, but also of their XWVDs. This means that the spectrum energy density of the sum of two signals does not reduce to the sum of the individual densities (unless the signals are spectrally disjoint). If $y_{1}\left(t\right)$ and $y_{2}\left(t\right)$ are mono-component signals, $WVD_{y_{1}}\left(t,\omega \right)$ and $WVD_{y_{2}}\left(t,\omega \right)$ are the auto-terms, while $2Re\{WVD_{y_{1}y_{2}}\left(t,\omega \right)\}$ is a cross-term.

**Figure 4:**
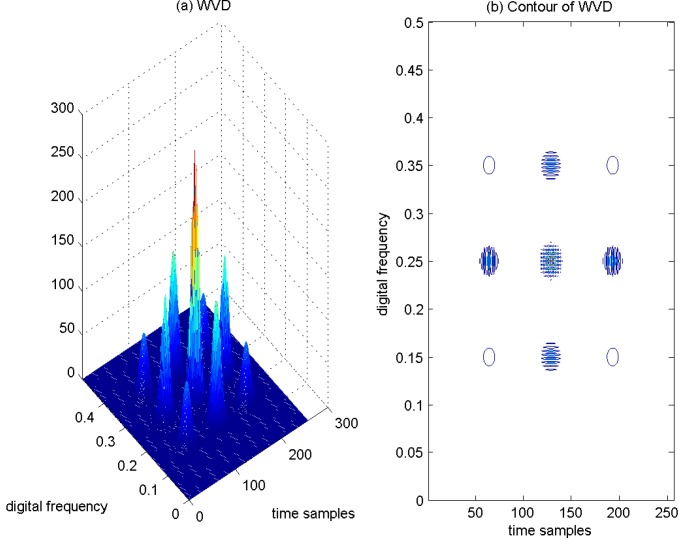
WVD of four Gaussian components’ signals. (**a**) WVD; (**b**) Contour of WVD.

As a result, if a signal contains more than one component, in the TF plane, its WVD suffers from spurious features containing cross-terms that occur halfway between each pair of auto-terms. The magnitude of these oscillatory cross-terms can be twice as large as the auto-terms, and they do not possess any physical meaning. The WVD of the aforementioned four Gaussian components’ signals is provided in Figure 4a, and correspondingly, the contour of the computed WVD is presented in Figure 4b. From Figure 4, there exist four peaks, which respectively denote the corresponding auto-terms of the four Gaussian components in the TF plane; and it is clear that the WVD method evidently provides better localization properties in the TF plane in comparison to the spectrogram approach. In addition, the WVD also presents six cross-terms, which occur between each pair of Gaussian signal components distributed in different time or frequency positions, and among them, two cross-terms overlap in the diagonal intersection point of the rectangle connected by the four vertices (*i.e.*, four Gaussian components in the TF plane). These extra cross-terms have large oscillating amplitudes due to the interaction of the different signal components. The magnitudes of the oscillatory cross-terms can be twice as large as the auto-terms; note that in the diagonal intersection position, the intensity of the corresponding cross-term can be four-times as large as the auto-terms, since there exist two cross-terms that overlap in the diagonal intersection point. In Figure 4b, the serious cross-terms are apparently present in the regions of the TF plane where we expect no energy at all, which makes a proper interpretation impossible. The presence of the cross-terms in the TF plane is the main drawback of the WVD approach.

## 4. Interference Detection Based on the Reassigned Spectrogram

From Section 3, the Heisenberg’s inequality prohibits the existence of the window function with an arbitrarily short time duration and small frequency bandwidth at the same time. Therefore, the conventional spectrogram approach presents the TF resolution trade-off problem and provides poor localization properties in the TF plane, which is not suitable to be directly used in interference detection for GNSS receivers. To deal with the TF resolution trade-off problem with the spectrogram, WVD can be usually used for detecting interference present in the received GNSS signal. Although the WVD has many good properties and provides nearly the optimal TF resolution that allows one to perfectly concentrate the signal components in the TF plane, due to its intrinsic quadratic nature, its use in practical applications is limited by the presence of serious oscillating cross-terms coexisting with the signal auto-terms, when it is applied to multi-component signals. These cross-terms may lead to an erroneous interpretation of the TF structure of the interfered GNSS signal, since they may overlap with the searched TF characteristics of the interfering signal. This drawback severely hinders the usefulness of WVD for identifying interference characteristics in the TF plane for GNSS receivers. In order to obtain a suitable trade-off between good cross-term attenuation and good TF concentration with the TF analysis, a reassigned spectrogram method has been proposed in interference detection for GNSS receivers.

### 4.1. Generalized TF Distribution

All of the existing TF distributions can be written in a generalized form, which can be used to facilitate the design of desirable TF transforms. This class of transform is known as Cohen’s class [11], and the definition of Cohen’s class of bilinear (or quadratic) TF distributions can be written as follows:

(28) $$\begin{array}{c} \begin{array}{cc} C(t,\omega ) & =\frac{1}{2\pi }\int_{-\infty }^{\infty }\int_{-\infty }^{\infty }AF\left(\tau ,\theta \right)g\left(\tau ,\theta \right)e^{-j(\theta t+\omega \tau )}\text{d}\theta \text{d}\tau \\ & =\frac{1}{2\pi }\int_{-\infty }^{\infty }\int_{-\infty }^{\infty }\int_{-\infty }^{\infty }y_{a}\left(s+\frac{\tau }{2}\right)y_{a}^{*}\left(s-\frac{\tau }{2}\right)g\left(\tau ,\theta \right)e^{-j[\theta (t-s)+\omega \tau ]}\text{d}s\text{d}\theta \text{d}\tau \end{array} \end{array}$$

where AF(τ,θ) is the ambiguity function defined in Equation (22); and g(τ,θ) is a two-dimensional parametrization function defined in the ambiguity function domain, which is called the kernel function of Cohen’s class. This kernel function determines the properties of the bilinear TF distribution, which is often a low-passing function and normally serves to mask out the interference in the original Wigner–Ville representation. When considering g(τ,θ)=1, WVD can be obtained.

In Cohen’s class, there exists a kernel formalism that allows one to interpret each element of the TF distribution as the convolution product of a central distribution (the WVD) with a parameterizing two-dimensional kernel. Therefore, Cohen’s class in Equation (28) can be rewritten as the two-dimensional convolution of the WVD of the signal $y_{a}\left(t\right)$ and a two-dimensional smoothing function, provided as follows:

(29) $$\begin{array}{c} \begin{array}{cc} C(t,\omega ;\Pi ) & =W_{y_{a}}\left(t,\omega \right)**\;\Pi \left(t,\omega \right)\\ & =\frac{1}{2\pi }\int_{-\infty }^{\infty }\int_{-\infty }^{\infty }\Pi \left(t-s,\omega -\theta \right)W_{y_{a}}\left(s,\theta \right)\text{d}s\text{d}\theta \end{array} \end{array}$$

(30) $$\begin{array}{c} \Pi \left(t,\omega \right)=\int_{-\infty }^{\infty }\int_{-\infty }^{\infty }g\left(\tau ,\theta \right)e^{-j(\omega \tau +\theta t)}\mathrm{d\tau d}\theta \end{array}$$

where (**) denotes the two-dimensional convolution operation, Π(t,ω) is a two-dimensional smoothing function and $W_{y_{a}}\left(s,\theta \right)$ is the WVD of the signal $y_{a}\left(t\right)$. Different TFRs can be obtained from the fundamental WVD by applying a different smoothing function Π(t,ω). Theoretically, these TF distributions suffer from a limitation inherent in the convolution product, since the revolutions of time and frequency are fixed, regardless of the local geometry of the signal signature. In many cases, especially when the TF readability is of primary importance, the kernel function Π(t,ω) is well designed to achieve some kind of TF smoothing; on the other hand, this smoothing leads to a less accurate TF localization of the signal components. Its shape and spread should be properly determined to achieve a suitable trade-off between good cross-term mitigation and good TF energy aggregation in the TF plane.

### 4.2. Reassignment Method

The most simple cross-term-free distribution of the Cohen’s class is the spectrogram, which can be expressed in Equation (29) using the smoothing kernel $\Pi \left(t,\omega \right)=W_{h}\left(t,\omega \right)$, where $W_{h}\left(t,\omega \right)$ denotes the WVD of the window function h(t). This smoothing operation removes the cross-terms present in the WVD $W_{y_{a}}\left(t,\omega \right)$ of the analyzed signal $y_{a}\left(t\right)$, but it also smears out the signal auto-terms. The mitigation of the cross-terms can be posed as a kernel function Π(t,ω) design problem subject to the constraint of preserving the good TF resolution of C(t,ω;Π). In order to improve the TF aggregation properties of C(t,ω;Π), the existing solution consists of a non-uniform post-processing of TF distributions guided by local signal characteristics, which can be implemented by the reassignment method. The principle of the reassignment method is to compensate the faults in mapping the TF distribution C(t,ω;Π) by relocating the value of the neighboring TF energy at its current location (t,ω) to the gravity center $\left(\overset{˰}{t},\overset{˰}{\omega }\right)$ rather than its geometrical center.

The starting point of the reassignment method is Equation (29). From Equation (29), we can know that the two-dimensional smoothing function Π(t−s,ω−θ) determines a certain TF region at the neighborhood nearby the point (t−s,ω−θ), inside which a weighted average of the WVD $W_{y_{a}}\left(s,\theta \right)$ of the signal $y_{a}\left(t\right)$ is performed. Cohen’s class C(t,ω;Π) is then the average of the TF energy located in a domain centered on (t,ω) and delimited by the essential support of Π. However, this mean value may not be symmetrically distributed around this geometrical center (t,ω); consequently, the geometrical center (t,ω) is not truly representative of such a TF region. In contrast, the TF energy gravity center of such a TF region is more approximate to represent the local energy distribution of the analyzed signal. The local TF energy distribution $\Pi \left(t-s,\omega -\theta \right)W_{y_{a}}\left(s,\theta \right)$ of Cohen’s class can be assumed as a distribution of mass, and it is better to assign the mass to the gravity center.

The TF energy averaging in Equation (29) leads to the attenuation of the oscillating cross-terms, but it also presents the undesirable effect of broadening the signal components in the TF plane. Therefore, the solution to avoid this effect is the adoption of the reassignment method, which can be generalized to all bilinear TF distributions. In order to improve the TF concentration of the signal components, the reassignment method is considered to relocate each value of Cohen’s class C(t,ω;Π) at any point (t,ω) to another point $\left(\overset{˰}{t},\overset{˰}{\omega }\right)$, which is the gravity center of the signal’s TF energy distribution around the point (t,ω).

According to Equation (29), the spectrogram in Equation (19) can be also expressed as a two-dimensional smoothing of the WVD, which can be obtained by using the smoothing kernel $\Pi \left(t,\omega \right)=W_{h}\left(t,\omega \right)$, written as follows:

(31) $$\begin{array}{c} \begin{array}{c} S\left(t,\omega \right)=\frac{1}{2\pi }\int_{-\infty }^{\infty }\int_{-\infty }^{\infty }W_{h}\left(t-s,\omega -\theta \right)W_{y_{a}}\left(s,\theta \right)\text{d}s\text{d}\theta \end{array} \end{array}$$

This above expression shows that the value of the spectrogram S(t,ω) at any point (t,ω) is a weighted sum of WVD $W_{y_{a}}\left(s,\theta \right)$ at the neighborhood nearby the point (t−s,ω−θ); thus, S(t,ω) is the sum of a whole TF energy distribution located around its geometrical center (t,ω). In order to avoid the resulting signal component broadening, an effective solution is to move this average from the point (t,ω), where S(t,ω) is computed for the center of gravity $\left(\overset{˰}{t},\overset{˰}{\omega }\right)$ of its composing energy distributions.

When the reassignment method is applied to the spectrogram in Equation (31), the obtained reassigned spectrogram can be written as follows:

(32) $$\begin{array}{c} S^{\left(r\right)}\left(t^{\prime },\omega^{\prime }\right)=\frac{1}{2\pi }\int_{-\infty }^{\infty }\int_{-\infty }^{\infty }S\left(t,\omega \right)\delta \left(t^{\prime }-\overset{˰}{t}\left(y_{a};t,\omega \right)\right)\delta \left(\omega^{\prime }-\overset{˰}{\omega }\left(y_{a};t,\omega \right)\right)\text{d}t\text{d}\omega \end{array}$$

where:

(33) $$\begin{array}{c} \overset{˰}{t}\left(y_{a};t,\omega \right)=\frac{\int_{-\infty }^{\infty }\int_{-\infty }^{\infty }s\Pi \left(t-s,\omega -\theta \right)W_{y_{a}}\left(s,\theta \right)\text{d}s\text{d}\theta }{\int_{-\infty }^{\infty }\int_{-\infty }^{\infty }\Pi \left(t-s,\omega -\theta \right)W_{y_{a}}\left(s,\theta \right)\text{d}s\text{d}\theta } \end{array}$$

(34) $$\begin{array}{c} \overset{˰}{\omega }\left(y_{a};t,\omega \right)=\frac{\int_{-\infty }^{\infty }\int_{-\infty }^{\infty }\theta \Pi \left(t-s,\omega -\theta \right)W_{y_{a}}\left(s,\theta \right)\text{d}s\text{d}\theta }{\int_{-\infty }^{\infty }\int_{-\infty }^{\infty }\Pi \left(t-s,\omega -\theta \right)W_{y_{a}}\left(s,\theta \right)\text{d}s\text{d}\theta } \end{array}$$

where $S^{\left(r\right)}\left(t^{\prime },\omega^{\prime }\right)$ is the spectrogram after reassignment; and δ(t) denotes the Dirac impulse.

The reassignment operation in Equation (32) leads to the construction of a modified version of the spectrogram, whose value at any point $\left(t^{\prime },\omega^{\prime }\right)$ is the sum of all of the TF energy distributions moved to this point. The most interesting property of the reassigned spectrogram is considered that the respective time and frequency reassigned location values $\overset{˰}{t}\left(y_{a};t,\omega \right)$ and $\overset{˰}{\omega }\left(y_{a};t,\omega \right)$ can also be derived from the partial derivatives of the phase of the STFT of $y_{a}\left(t\right)$, written as follows:

(35) $$\begin{array}{c} \overset{˰}{t}\left(y_{a};t,\omega \right)=-\frac{\partial \Phi_{y_{a}}\left(t,\omega \right)}{\partial \omega } \end{array}$$

(36) $$\begin{array}{c} \overset{˰}{\omega }\left(y_{a};t,\omega \right)=\omega +\frac{\partial \Phi_{y_{a}}\left(t,\omega \right)}{\partial t} \end{array}$$

where $\Phi_{y_{a}}\left(t,\omega \right)$ is the phase of the STFT of the analyzed signal $y_{a}\left(t\right)$, i.e.,$\Phi_{y_{a}}\left(t,\omega \right)=arg\left(STFT_{y_{a}}\left(t,\omega \right)\right)$. The above-mentioned expressions in Equations (35) and (36) can be interpreted respectively as the local group delay and the local instantaneous frequency of the signal $y_{a}\left(t\right)$ observed in the TF domain imposed by the analysis window h(t).

Although the expressions in Equations (35) and (36) are physically meaningful, unfortunately, they do not lead to an efficient implementation of the reassignment operation for discrete time signals, since the derivatives should be approximated by first order differences. Thus, the calculation method for the reassignment operators remains unused despite its attractive theoretical background. In practice, a more efficient implementation of the calculation of reassigned location can be obtained, written as follows:

(37) t˰(ya;t,ω)=t−ReSTFTya(t,ω;th)STFTya*(t,ω;h)|STFTya(t,ω;h)|2

(38) ω˰(ya;t,ω)=ω+ImSTFTya(t,ω;Dh)STFTya*(t,ω;h)|STFTya(t,ω;h)|2

where Re and Im represent real and imaginary parts, respectively; $t_{h}=th\left(t\right)$ means the time ramped smoothing window multiplied by the time variable *t*; $D_{h}\left(t\right)=dh(t)/dt$ denotes the smoothing window using the first time derivative of the window function h(t); and * indicates a complex conjugate. Explicit phase differentiations can be avoided by computing two additional STFTs $STFT_{y_{a}}\left(t,\omega ;t_{h}\right)$ and $STFT_{y_{a}}\left(t,\omega ;D_{h}\right)$. These new expressions in Equations (37) and (38) do not focus any more on the phase of the STFT, and for computational simplicity, the reassigned point $\left(\overset{˰}{t}\left(y_{a};t,\omega \right),\overset{˰}{\omega }\left(y_{a};t,\omega \right)\right)$ is computed from the ratios of the STFTs rather than from the phase derivatives.

**Figure 5:**
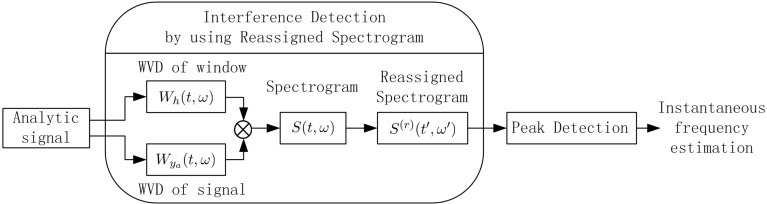
Interference detection by using the reassigned spectrogram for GNSS receivers.

The reassigned spectrogram $S^{\left(r\right)}\left(t^{\prime },\omega^{\prime }\right)$ can be adopted to improve TF energy aggregation properties in the TF plane, and it is also free of cross-term artifacts; therefore, in this work, the TF distribution by using the reassigned spectrogram has been proposed in interference detection for GNSS receivers, which is clearly illustrated in Figure 5. The two-dimensional convolution of the WVD $W_{y_{a}}\left(t,\omega \right)$ of the analytical signal $y_{a}\left(t\right)$ with the WVD $W_{h}\left(t,\omega \right)$ of the window function h(t) (i.e., the two-dimensional smoothing kernel $\Pi \left(t,\omega \right)=W_{h}\left(t,\omega \right)$) is performed to obtain the spectrogram S(t,ω); after the reassignment is performed in S(t,ω), the obtained reassigned spectrogram $S^{\left(r\right)}\left(t^{\prime },\omega^{\prime }\right)$ has good TF readability, which is suitable for multi-component signal analysis. Therefore, the proposed TF analysis by using the reassigned spectrogram is able to clearly differentiate the spectral characteristic of the interfering signal from that of the useful GNSS signal; thus, the instantaneous frequency of the disturbing term in the interfered GNSS signals can be effectively estimated by detecting the peaks of the reassigned spectrogram.

## 5. Performance Evaluation

In this section, the performance of the proposed interference detection method based on the TF analysis by using the reassigned spectrogram is analyzed. In particular, this proposed algorithm, which adopts the reassigned spectrogram to detect sweep interference in GNSS receivers, is compared to the conventional interference detection approaches in the disturbing scenario.

**Figure 6:**
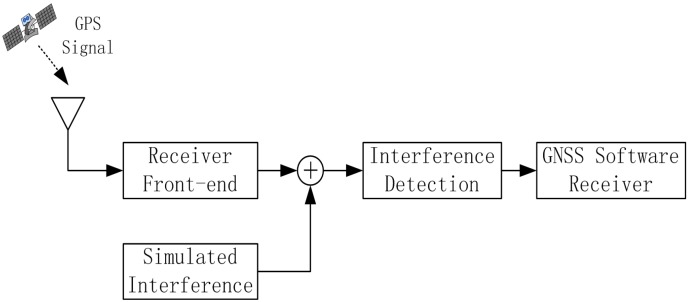
Scheme of the measurement test in the lab.

**Figure 7:**
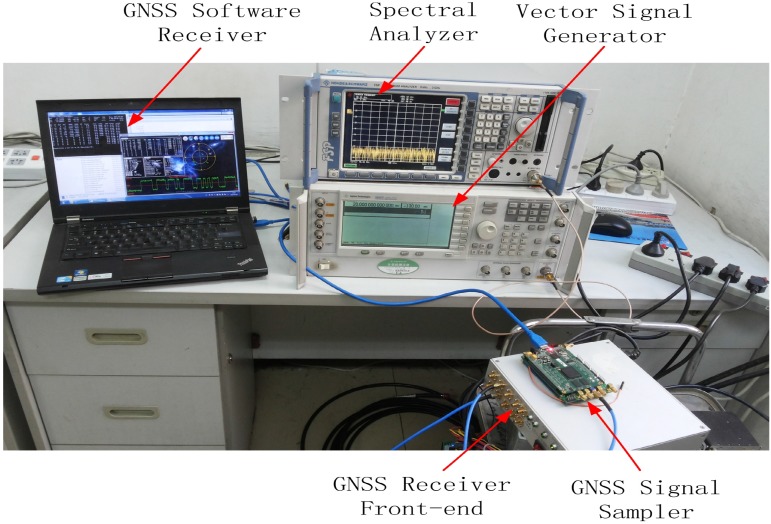
Experimental setup of the measurement test in the lab.

The mentioned interference detection approaches are tested on real GPS data collected by using the GNSS software receiver developed at Beihang University, China [24]. The scheme of the test is reported in Figure 6, while an image of the experimental setup adopted for collecting the GPS data corrupted by sweep interference is depicted in Figure 7. The real GPS samples are collected by using the GNSS software receiver connected to the Trimble Zephyr Geodetic 2 antenna placed on the roof of the new main building at Beihang University in an open-sky static condition; the software interferer is adopted for generating the sweep interfering signal for GNSS applications. The generated sweep interference is added to the GPS samples collected by the GNSS receiver front-end.

In the experiment, the scenario adopted for the test is characterized by the setting parameters listed in Table 1, representing the real GPS L1 signal in zero mean Gaussian noise corrupted by a constant amplitude linear frequency-modulated interference (linear chirp), which is commonly considered as a test bench in interference detection for GNSS receivers.

**Table 1:** Experimental setting parameters.

Parameter	Value
Sampling frequency, $f_{s}$	24 MHz
Intermediate frequency, $f_{IF}$	40.42 MHz
Code length	1023 chips
Sweep period	1 ms
Spectrogram analysis window	Hamming

In the experiment settings, the carrier power to noise power density ratio $C/N_{0}$ is 46 dB-Hz, and the JNR value of the sweep interference is set to be −2 dB; in a linear chirp, the instantaneous frequency $f_{inst}$ of the interfering signal evolves linearly with time over the interval $\left[f_{L1}+\Delta f_{0},f_{L1}+\Delta f_{1}\right]$, where $f_{L1}$ is the GPS L1 signal center frequency, $\Delta f_{0}=+5\;MHz$ and $\Delta f_{1}=-7\;MHz$.

**Figure 8:**
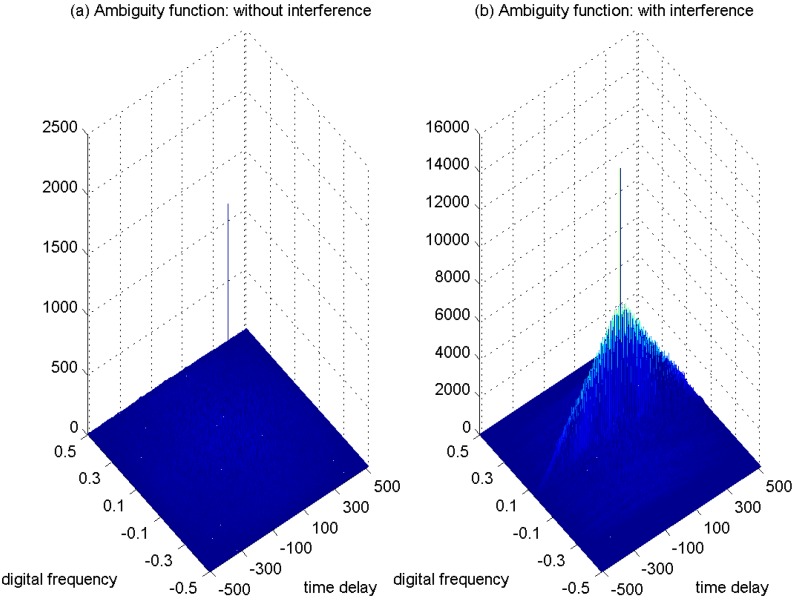
Ambiguity function of the GPS L1 signal. $C/N_{0}$ = 46 dB-Hz. (**a**) Without interference; (**b**) With interference, JNR = −2 dB.

In Figure 8a, the ambiguity function of the GPS L1 signal without interference is shown as a spike. In the case with the presence of sweep interference, the ambiguity function of the interfered GPS L1 signal is depicted in Figure 8b, where the disturbing effect can be clearly observed. The adoption of the ambiguity function is beneficial to better monitor the interference contribution in the interfered GPS L1 signals.

**Figure 9:**
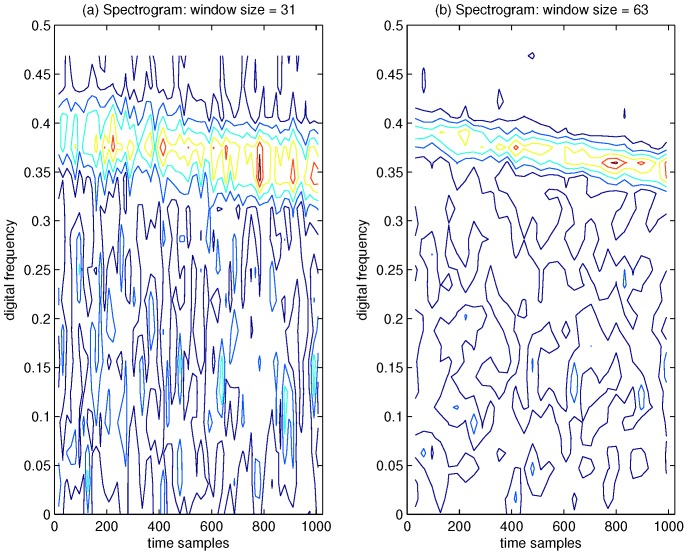
Spectrogram of the GPS L1 signal with sweep interference. The spectrogram has been evaluated by using a Hamming window. $C/N_{0}$ = 46 dB-Hz, JNR = −2 dB. (**a**) Window size = 31; (**b**) Window size = 63.

In Figure 9, the spectrogram of the GPS L1 signal with sweep interference is depicted, where the Hamming window function is chosen. In Figure 9a, the window size is 31 samples, where the disturbing term more or less emerges in the TF plane, but very poor TF localization properties are obtained. In order to evaluate the TF characteristics of the spectrogram by increasing the length of the analysis window, the spectrogram of the interfered GPS L1 signal is presented in Figure 9b, where the window length is increased to 63 samples. From Figure 9, it is clear that a long time window leads to improved frequency resolution and inevitably yields poor resolution in time. The spectrogram is nonlinear, but this nonlinearity results from the operation of the squared magnitude and, therefore, does not lead to undesirable cross-terms in the TF distribution.

**Figure 10:**
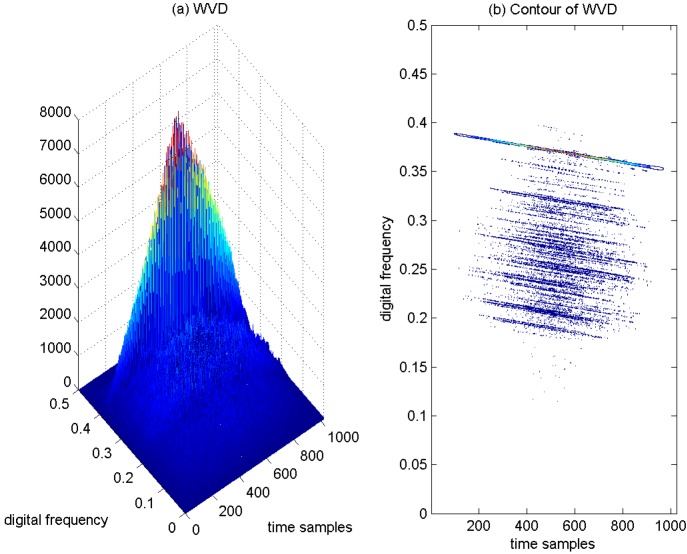
WVD of the GPS L1 signal with sweep interference. $C/N_{0}$ = 46 dB-Hz, JNR = −2 dB. (**a**) WVD; (**b**) Contour of WVD.

In order to deal with the TF resolution trade-off problem with the spectrogram approach, the WVD of the GPS L1 signal with the sweep interference is depicted in Figure 10a, and correspondingly, the contour of the computed WVD is presented in Figure 10b. The interfering term presents a linear behavior in frequency, which is well localized in the restricted area of the TF plane. Due to the interaction of the different components in the interfered GPS L1 signals, the presence of serious cross-terms is apparently observed in Figure 10b, which makes interpretation quite difficult and brings much error to the estimation of the instantaneous frequency of the interfering signal. The price paid for good TF resolution with the WVD approach is the undesirable cross-terms present in the TF plane.

**Figure 11:**
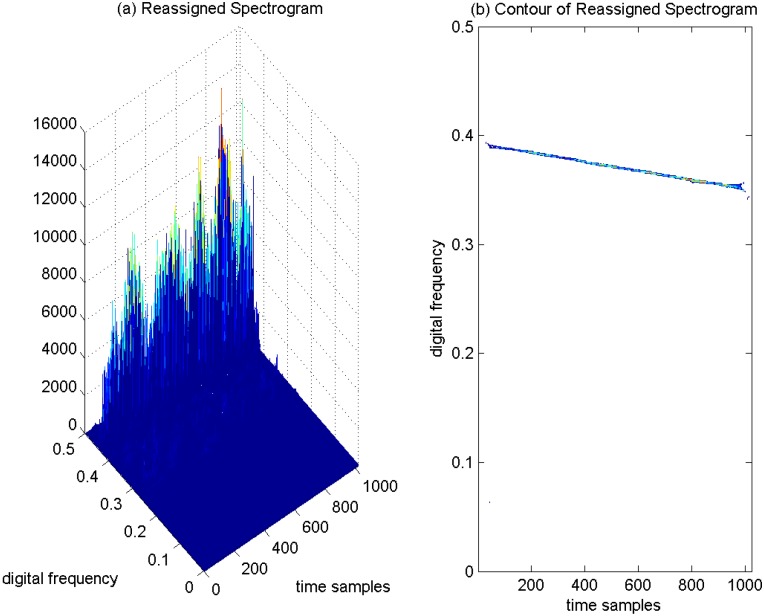
Reassigned Spectrogram of the GPS L1 signal with sweep interference. $C/N_{0}$ = 46 dB-Hz and JNR = −2 dB. (**a**) Reassigned Spectrogram; (**b**) Contour of Reassigned Spectrogram.

Therefore, in order to improve the TF energy concentration properties and to avoid the cross-terms present in the quadratic TF distribution at the same time, a novel TF distribution by using the reassigned spectrogram has been proposed in interference detection for GNSS receivers. The reassigned spectrogram of the interfered GPS L1 signal is depicted in Figure 11a, and the corresponding contour of the reassigned spectrogram is provided in Figure 11b. In Figure 11a, it is clear that the proposed reassigned spectrogram yields very sharp peaks in the TF plane denoting the undesired sweep interfering term in the interfered GPS L1 signal; accordingly, in Figure 11b, in the TF plane, there exists a very strict line representing the instantaneous frequency of the sweep interfering signal by using the reassigned spectrogram method, which provides representations that are easy to interpret in interference detection for GNSS receivers. The reassigned spectrogram squeezes the TF energy distributions concentrated at the instantaneous frequency line in the TF plane, where the TF energy density components are apparently strengthened. The reassigned spectrogram greatly improves the sharpness of the ridges of the sweep interfering term, which are highly localized at the instantaneous frequency line in the TF plane, since the spread energy of the spectrogram is relocated from the geometrical center to the gravity center in the TF energy distribution. Therefore, the instantaneous frequency of each component of the sweep interfering signal can be revealed by detecting the local energy peaks in the reassigned spectrogram. The reassigned spectrogram makes the TF energy of the sweep interference concentrate at the instantaneous frequency line, which presents good readability in the TF plane. Much improved TF aggregation properties can be achieved in comparison to the conventional spectrogram approach; and meanwhile, the reassigned spectrogram is free of cross-term artifacts during the TF analysis; this is its advantage when compared with the WVD approach.

This proposed TF analysis based on the reassigned spectrogram solves the cross-term problem of the bilinear TF distribution and keeps good TF resolution in the TF plane, which has been proven to be valid and effective to adopt in interference detection for GNSS receivers in jamming environments. With this developed technique, the interfering signal can be correctly identified and its instantaneous frequency can be precisely estimated. The estimated instantaneous frequency of the interfering signal can be used in the notch filter, which is used in the interference mitigation unit of the GNSS receiver for the removal of the interfering signal.

**Figure 12:**
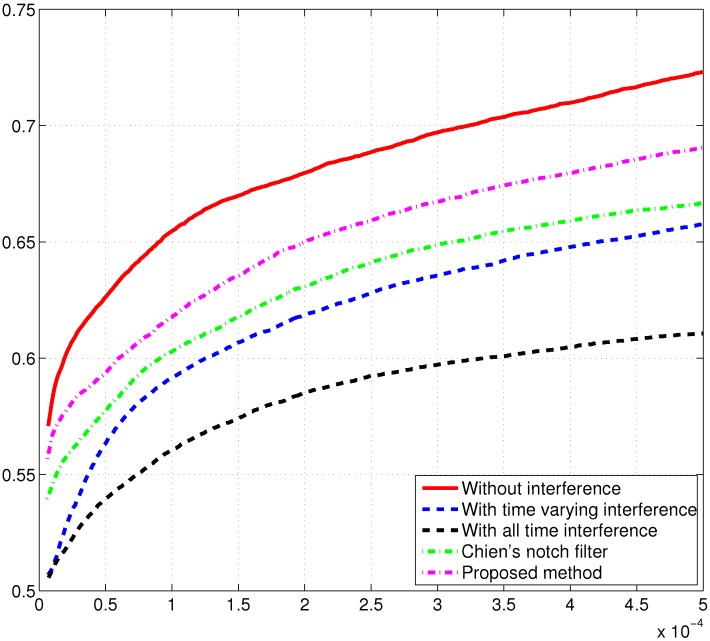
ROC curves of the GPS L1 signal in different signal scenarios. The coherent integration time is 2 ms, $C/N_{0}$ = 35 dB-Hz.

In order to analyze the performance of the interference mitigation unit and, in particular, the impact of a notch IIR filter adopted in the GNSS receiver, ROC curves under different signal scenarios have been evaluated by Monte Carlo simulations. In Figure 12, the estimated instantaneous frequency of the interference is used to down-convert the sweep interfering signal, where the $C/N_{0}$ value is set to be 35 dB-Hz, the JNR value is set to be 10 dB when the sweep interference is present in the received GPS L1 signal, and the coherent integration time is set to be 2 ms in the acquisition process by using the parallel acquisition scheme in the time domain [20,21]. In the simulation, two typical disturbing signal cases are considered: the all-time interference present in the received GPS L1 signal and the time-varying interference alternately appearing in one GPS C/Acode period (1 ms) and disappearing in the other GPS C/A code period, since the coherent integration time is chosen as 2 ms in the acquisition process. From Figure 12, it is easy to find that the ROC curve in absence of interference shows ideal acquisition performance; when no interference mitigation approach is applied, the presence of the sweep interference in the received GPS L1 signal results in serious acquisition performance degradation, and in detail, as expected, the acquisition performance obtained in the case of all-time interference is clearly worse than that achieved in the time-varying interference case.

Recently, Chien developed a notch IIR filter with a lattice structure to mitigate the interfering signal for GNSS receivers [25]. This algorithm suggested using the second-order statistical value of the notch frequency-related parameter *β* to detect the interfering signal. In our proposed interference detection and mitigation method, the interfering signal is detected by the reassigned spectrogram and then mitigated by the notch IIR filter for GNSS receivers. In order to evaluate the interference detection and mitigation capability, our proposed method is compared to the state-of-the-art Chien’s notch filter. In the interference mitigation performance comparison, we assume that the sweep interference is always present in the received GPS L1 signal. As shown in Figure 12, enhanced acquisition performance can be obtained by using Chien’s notch filter when compared to the aforementioned two cases of no interference rejection. On the other hand, Figure 12 indicates that our proposed method provides much improved acquisition performance in comparison to Chien’s notch filter, which further verifies the validity and effectiveness of the proposed anti-interference algorithm for GNSS receivers in jamming environments.

## 6. Conclusions

In this paper, a novel TF analysis method based on a reassigned spectrogram has been proposed to detect sweep interference for GNSS receivers. In order to prove the advantages and effectiveness of the developed technique, a comprehensive performance comparison has been carried out with the existed spectrogram and WVD approaches. The experiments have been performed on GPS L1 signals in the disturbing scenario in order to support the theoretical analysis among the aforementioned TF distributions used in interference detection for GNSS receivers.

From the analysis results, the spectrogram approach presents the TF resolution trade-off problem and provides poor localization properties in the TF plane, since the analysis window is used, and in practice, it is not suitable to be used for detecting the disturbing term in the interfered GNSS signals; the WVD approach shows good TF resolution in identifying the characteristics of the interfering signal, but it presents a very severe cross-term problem due to the interaction of different signal components, which makes proper disturbance interpretation impossible.

In order to eliminate the cross-terms and to preserve good time and frequency resolutions in the TF plane at the same time, a new TF distribution by using the reassigned spectrogram has been proposed in interference detection for GNSS receivers. This proposed technique efficiently combines the removal of the cross-terms provided by the spectrogram itself according to its inherent nature and an increased TF concentration of the auto-terms of the signal components achieved by the reassignment method. From the analysis results, the proposed interference detection method based on the reassigned spectrogram successfully solves the cross-term problem and, meanwhile, provides good readability and TF localization properties, showing much improved interference detection performance in comparison to the state-of-the-art TF analysis approaches.

Moreover, the notch IIR filter has been adopted in the interference mitigation unit of the GNSS receiver providing significantly improved acquisition performance in terms of ROC curves in the presence of sweep interference in the received GNSS signals. The use of the notch IIR filter in the GNSS receiver has been proven to be valid and effective to improve the interference mitigation performance in jamming environments.

The developed interference detection and mitigation techniques are very promising to be adopted in the design of an anti-interference GNSS receiver especially for civil aviation and military applications.
